# Benign giant mediastinal schwannoma presenting as cardiac tamponade in a woman: a case report

**DOI:** 10.1186/1752-1947-5-61

**Published:** 2011-02-14

**Authors:** Motoyasu Kato, Satomi Shiota, Kazuo Shiga, Haruhi Takagi, Hiroaki Mori, Mitsuaki Sekiya, Kenji Suzuki, Toshimasa Uekusa, Kazuhisa Takahashi

**Affiliations:** 1 Department of Respiratory Medicine, School of Medicine, Tokyo, Japan; 2 Department of General Thoracic Surgery Juntendo University, School of Medicine, Tokyo, Japan; 3 Department of Pathology Kanto Rosa Hospital, Kanagawa, Japan

## Abstract

**Introduction:**

Mediastinal schwannomas are typically benign and asymptomatic, and generally present no immediate risks. We encountered a rare case of a giant benign posterior mediastinal schwannoma, complicated by life-threatening cardiac tamponade.

**Case presentation:**

We report the case of a 72-year-old Japanese woman, who presented with cardiogenic shock. Computed tomography of the chest revealed a posterior mediastinal mass 150 cm in diameter, with pericardial effusion. The cardiac tamponade was treated with prompt pericardial fluid drainage. A biopsy was taken from the mass, and after histological examination, it was diagnosed as a benign schwannoma, a well-encapsulated non-infiltrating tumor, originating from the intrathoracic vagus nerve. It was successfully excised, restoring normal cardiac function.

**Conclusion:**

Our case suggests that giant mediastinal schwannomas, although generally benign and asymptomatic, should be excised upon discovery to prevent the development of life-threatening cardiopulmonary complications.

## Introduction

Mediastinal schwannomas are typically benign and asymptomatic, and generally present no immediate risks. We encountered a rare case of a giant benign posterior mediastinal schwannoma that was complicated by life-threatening cardiac tamponade.

## Case presentation

A 72-year-old Japanese woman presented at the emergency room with cardiogenic shock and hypoxia. She reported the presence of exercise-induced dyspnea and right chest pain for several weeks. Her history included discovery two years previously of a posterior mediastinal tumor, 130 mm in diameter; because she was asymptomatic, our patient had declined further detailed examination and treatment at the time.

On physical examination, we found our patient's skin to be diaphoretic. She had a systolic blood pressure of 80 mmHg by palpation, atrial fibrillation with a heart rate of 130 beats/minute, and no detectable paradoxical pulse. She had severe hypoxia with an oxygen saturation level of 85% on 100% oxygen at 10 litres/min. Cardiogenic shock was strongly suspected.

**Figure 1:**
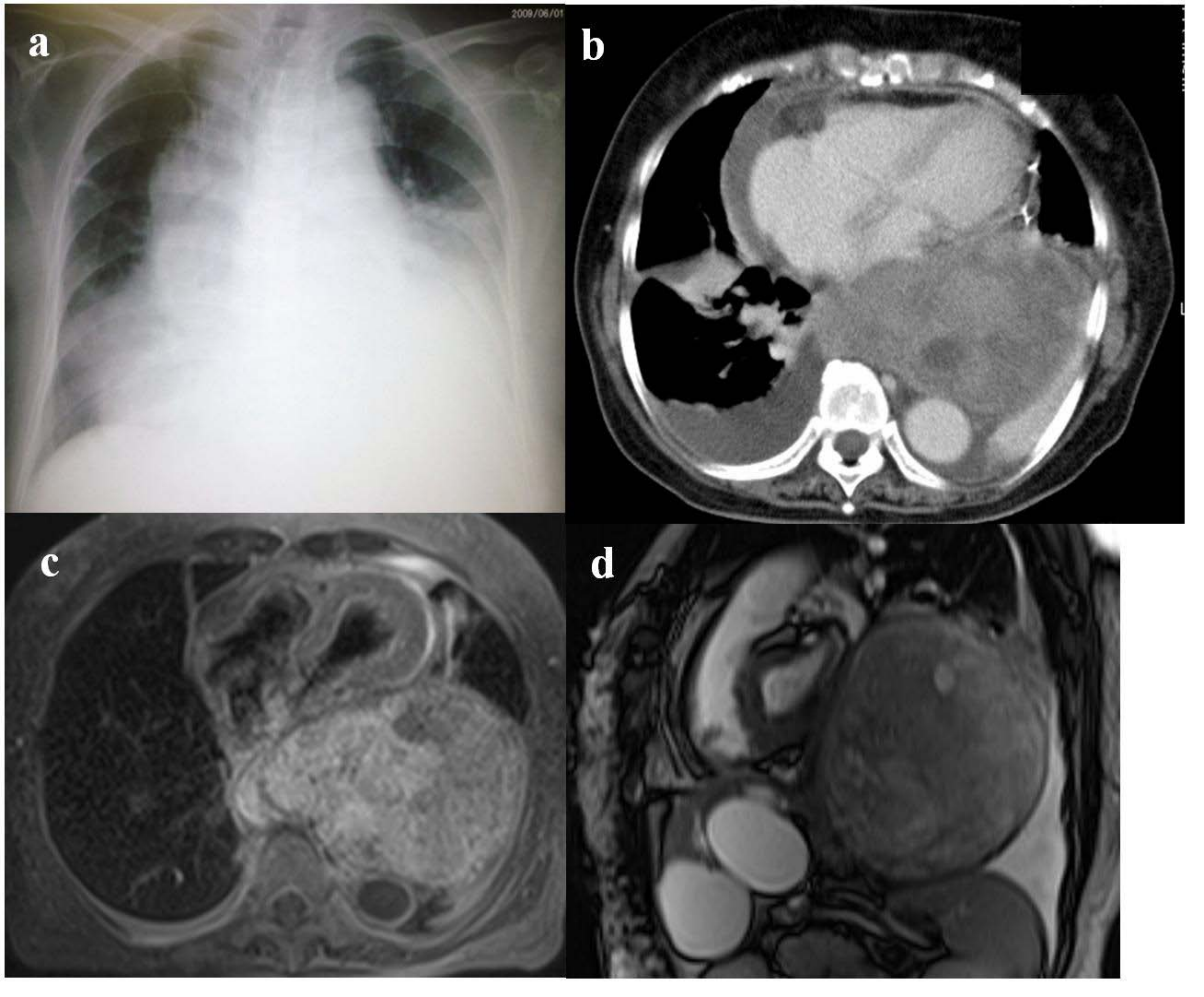
**Lung imaging.** (a) Chest radiograph obtained on the day of hospital admission showing bilateral pleural effusion, mediastinal widening and cardiac enlargement. (b) Contrast-enhanced chest computed tomography image (lung window) taken during pericardial drainage, showing a giant posterior mediastinal tumor, pericardial effusion and bilateral pleural effusion. (c) Transversal T1-weighted magnetic resonance imaging (MRI) scan of the chest taken after pericardial drainage, showing a giant encapsulating tumor in the posterior mediastinum compressing the heart. (d) Sagittal True SSFP (steady state free precession) MRI image, showing the tumor occupies most of left thoracic cavity.

Laboratory values for blood coagulation and blood cell counts were normal. Chest radiography showed an enlarged heart with hypolucent areas in both lung fields, and bilateral pleural effusion (Figure 1a). Computed tomography of the chest revealed a sharply marginated tumor, 150 mm in size, in the posterior mediastinum, and pericardial effusion (Figure 1b). Echocardiography showed a large pericardial effusion with diastolic collapse of the right side of the heart, indicating cardiac tamponade, and prompting us to carry out pericardial drainage.

A total of 1200 ml of cloudy fluid was aspirated, which was analyzed and found to contain 4.8 g/dl of total protein, 3941 cells/μl, with an erythrocyte volume fraction of 11.6%, implying that it was exudate fluid. Lymphocytes were the dominant cell type without any evidence of malignant cells. The fluid was classified as class II cytology. Bacterial and fungal cultures produced no growth.

A transcutaneous ultrasound-guided biopsy of the tumor was obtained, and the histological findings were consistent with a benign schwannoma. Magnetic resonance imaging of the chest after pericardial drainage showed that the tumor occupied the posterior mediastinum of the left pleural cavity, and was clearly separated from the cardiac structures (Figure 1c,d).

**Figure 2:**
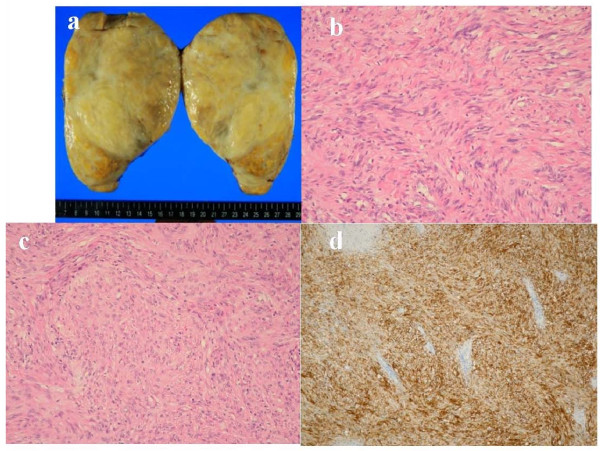
**Macro and Histological images. **(**a**) Macro findings of an encapsulated solid tumor measuring 140 mm at its the greatest dimension. The cut surface was smooth with a pale yellow color, and showed numerous mucinous foci without extracapsular invasion. Microscopically, the tumor exhibited (**b**) Antoni A areas composed of ill-defined fascicles of spindle cells and (**c**) loosely organized Antoni B areas. (**d**) Immunohistochemically, the tumor cells were strongly positive for S-100 protein.

Complete surgical resection was carried out using a left thoracotomy approach, and the mass was found to be a giant tumor, 140 × 100 mm in size, originating from the vagus nerve(Figure 2a). Although adhering broadly to part of the parietal epicardium, lower left lobe of lung, diaphragm and descending aorta, and being fully decorticated, the well encapsulated tumor had not infiltrated the adjacent organs or the pericardium. Histological examination showed that the tumor was consistent with a benign schwannoma, characterized by a proliferation of spindle cells with cellular uniformity and immunoreactivity to S-100 protein (Figure 2d). Our patient was discharged on the sixth postoperative day, and had an uneventful recovery.

## Discussion

This was a rare case of a benign posterior mediastinal schwannoma, originating from the vagus nerve, complicated by life-threatening cardiac tamponade. Schwannomas are benign nerve sheath neoplasms of Schwann cell origin, and are the most common of the neurogenic mediastinal tumors. Nearly 45% of schwannomas occur in the head and neck, with 9% occurring in the mediastinum [1]. Generally, mediastinal schwannomas are slow-growing and asymptomatic and rarely degenerate into malignant tumors. The origin of our patient's tumor is atypical in that it stemmed from the intrathoracic branches of the vagus nerve. Mediastinal schwannomas most frequently arise in a paravertebral location from sympathetic trunks or intercostal nerves [2,3]. Schwannomas originating from the vagus nerve within the mediastinum are rare, comprising only 1.4% of intrathoracic schwannomas [4].

The hemodynamic clinical course of our patient was also unusual, as there are few reported cases of schwannomas with cardiac involvement. Two cases of benign tumors in the ventricle epicardium involving pericardial effusion have been described [5,6], and two further cases were reported as malignant intrapericardial schwannomas with cardiac tamponade [7,8].

To the best of our knowledge, this is the first reported case of a benign extrapericardial mediastinal schwannoma presenting with life-threatening cardiac tamponade caused by a large volume of pericardial effusion. Previous reported cases of malignant meidiastinal schwannoma presenting as cardiac tamponade in Recklinghausen's disease had a rapidly fatal outcome after pericardial drainage, whereas in our case, drainage produced rapid restoration of normal cardiac function as measured by echocardiography, with no recurrent fluid accumulation after complete excision of the tumor,.

Cardiac tamponade can be induced by either slow or rapid accumulation of pericardial fluid. With slow accumulation, the volume can become quite large and still cause no symptoms [9,10]. Thus, we believe that the fluid accumulation in our patient developed over a long period of time, and that the cardiac tamponade was not caused by the tumor directly infiltrating or perforating the cardiac tissue. Our patient was not anemic on admission, indicating no significant hemorrhage during her clinical course. In the case of chronic idiopathic pericardial effusion, fibrosis or inflammatory cell infiltration has been reported [11] as a change in pericardial histology. In our patient, the chronic pericardial inflammation induced by the giant mediastinal schwannoma occupying the posterior mediastinum of the left pleural cavity might have induced similar histological changes and thickening of the pericardium. It is possible that the thickened pericardium impairs fluid re-absorption, and the high colloid osmotic pressure of the pericardium fluid increases the tendency to fluid accumulation,

Although giant mediastinal schwannomas are usually benign, this case suggests that they can become life-threatening and thus should be aggressively and completely resected once discovered to prevent cardiopulmonary complications. Likewise, in cases of cardiac emergencies, the possible presence of large benign mediastinal schwannomas or other tumors should be investigated.

## Conclusions

In conclusion, we report a rare case of a giant benign posterior mediastinal schwannoma, originating from the vagus nerve, presenting with life-threatening cardiopulmonary complications of significant pericardial effusion leading to cardiac tamponade. Immediate drainage and complete surgical excision of the tumor successfully restored normal cardiac function and hemodynamic surgical intervention of benign giant posterior mediastinal schwannomas is recommended in similar cases to prevent the occurrence of cardiac tamponade.

## Competing interests

The authors declare that they have no competing interests.

## Consent

Written informed consent was obtained from our patient for publication of this case report and any accompanying images. A copy of the written consent is available for review by the Editor-in-Chief of this journal.

## Authors' contributions

MK and SS reviewed the clinical data and were major contributors in writing the manuscript. KS, HT, HM and KT were involved with patient management. MS performed the histological examination of the biopsy. KS was our patient's attending surgeon and provided information on our patient. TU analyzed histological data and performed the immunohistochemical analysis. All authors read and approved the final manuscript.
